# Sensorimotor rhythm and muscle activity in patients with stroke using mobile serious games to assist upper extremity rehabilitation

**DOI:** 10.3389/fresc.2023.1234216

**Published:** 2023-11-17

**Authors:** Zihe Chen, Tingmin Yan, Jinchun Wu, Yixuan Liu, Chunyun Zhang, Tianjian Cui

**Affiliations:** ^1^ School of Art, Southeast University, Nanjing, China; ^2^ School of Mechanical Engineering, Southeast University, Nanjing, China; ^3^ Department of Neurosurgery, First Hospital of Jilin University, Changchun, China

**Keywords:** upper limb hemiplegia, serious games, stroke, rehabilitation, electroencephalogram, electromyography, event-related desynchronization

## Abstract

**Introduction:**

Exercise rehabilitation is crucial for neurological recovery in hemiplegia-induced upper limb dysfunction. Technology-assisted cortical activation in sensorimotor areas has shown potential for restoring motor function. This study assessed the feasibility of mobile serious games for stroke patients' motor rehabilitation.

**Methods:**

A dedicated mobile application targeted shoulder, elbow, and wrist training. Twelve stroke survivors attempted a motor task under two conditions: serious mobile game-assisted and conventional rehabilitation. Electroencephalography and electromyography measured the therapy effects.

**Results:**

Patients undergoing game-assisted rehabilitation showed stronger event-related desynchronization (ERD) in the contralateral hemisphere's motor perception areas compared to conventional rehabilitation (*p* < 0.05). RMS was notably higher in game-assisted rehabilitation, particularly in shoulder training (*p* < 0.05).

**Discussion:**

Serious mobile game rehabilitation activated the motor cortex without directly improving muscle activity. This suggests its potential in neurological recovery for stroke patients.

## 1. Introduction

Stroke is the leading cause of disability in adults (1), and patients with stroke often suffer from mild upper limb (UL) hemiplegia (2). Notably, few individuals achieve full recovery of the UL at 6 months after stroke (3). Rehabilitation for somatosensory impairment focuses on restoring the affected neuromuscular functions and achieving independent body control (4). Owing to the plasticity of the brain, even simple movements can activate cortical tissue in the sensorimotor areas of the brain, which can partially or fully restore impaired motor function (5).

Electroencephalogram (EEG) and electromyography (EMG) signals are used for detecting and evaluating rehabilitation in stroke survivors. Attempting motor tasks is one of the most commonly used experimental paradigms for EEG (6). The specific EEG rhythm of the cortex in the sensorimotor area of the EEG-based brain–computer interface system can assess the brain activation degree (7). This can be assessed by measuring the event-related desynchronization (ERD) or event-related synchronization (ERS) of alpha and beta rhythms that occur in the sensorimotor cortex. EMG detects electrical signals generated by nerve-activated muscles (8) to assess muscle function in patients undergoing rehabilitation.

Rehabilitation interventions for UL aim to improve activities of daily living, functional independence, and quality of life (9). Studying traditional rehabilitation interventions has shown that an early intensive practice of active functional tasks, usually based on grasping, tapping, and reaching, results in more positive outcomes in upper-extremity rehabilitation (10). However, repetitive rehabilitation tasks (11) and therapist rehabilitation interactions (12) have been found to not significantly improve outcomes.

For the rehabilitation of stroke survivors, technology has potential therapeutic benefits and offers interesting and effective adjuncts. In multiple studies, the use of novel rehabilitation interventions such as UL rehabilitation robots (13), virtual reality systems (14), and other technologies has been confirmed to be beneficial for patients with UL hemiplegia. Technological assistance plays an auxiliary role in injury rehabilitation. To enable patients to perform rehabilitation exercises without restriction in time and place and obtain immediate feedback on the quality of their performance (15), mobile applications (apps) have become an emerging tool in the field of rehabilitation intervention research because of their mobility, multifunctionality (such as reminders, interactions, and feedback), and ability to empower patients with autonomous treatment (16). Furthermore, apps can often make playful interventions in the form of serious games. Serious games have rehabilitation as their primary goal while combining entertainment, attentional engagement, and problem-solving abilities (17). In addition, they conform to the multimotor relearning principles that form the basis of effective neurorehabilitation interventions (18). Researchers have developed a serious game that provides UL rehabilitation training for patients with stroke. The app captures the movement of the patient through the integrated sensor of the smartphone and visualizes the limb movements detected by the mobile phone using different depictions (15).

We aimed to study the effects of serious gaming using smartphones on the motor rehabilitation of patients with stroke. In this experiment, we set up two groups of controlled rehabilitation conditions, namely, game-assisted and conventional rehabilitation. Each patient participated in two experiments, which were conducted on two random days under different rehabilitation conditions to minimize possible training effects. The UL rehabilitation content sets three items, namely, shoulder training (a), elbow training (b), and wrist training (c). Each of this rehabilitation training content is divided into several motor tasks and monitors the brain and muscle activity of the patients in real time during the experiment. The results were obtained by comparing ERD/ERS and other data to analyze the sensorimotor rhythm of the same patient under the rehabilitation conditions of the two groups. The related muscle activity intensity was analyzed by comparing MF/RMS and other data. We hypothesized that the brains and motor muscles of patients would respond better to MA tasks during rehabilitation activities assisted by serious mobile games than without app use.

## 2. Materials and methods

### 2.1. Equipment and materials

In the experimental setup, we used a smartphone that measures 6.1 inches and weighs 218 g. It is equipped with an internal accelerometer, gyroscope, linear motor, and speaker. The dedicated serious game includes three intervention modules focusing on shoulder, elbow, and wrist rehabilitation. The patients engage in rehabilitation courses while holding the smartphone. The team meticulously designed the app interface, which incorporates the following distinctive features. (1) Vibration feedback: The app utilized vibration feedback to enhance the sensory perception of the limbs of the patients. The intensity of the vibrations guided the patients in performing standardized rehabilitation movements. (2) Voice guidance: The app provided real-time audio instructions and rhythmic cues for each step of the exercises. (3) Motion tracking: The app utilized the accelerometer and gyroscope to monitor and record the trajectory of each movement. After each rehabilitation session, the recorded data were visually presented on the interface to motivate the patients in their rehabilitation efforts.

Based on a systematic review and meta-analysis conducted by Veerbeek et al. (19) in 2014, the most robust evidence supporting optimal interventions for promoting UL recovery after stroke involves strengthening, repetition, and task-oriented exercises. The Graded Repetitive Arm Supplementary Program (GRASP) is a self-management intervention designed for the paralyzed UL, following these evidence-based principles. A study in 2009 found that the GRASP was effective in enhancing UL function and usage during inpatient rehabilitation and has been widely implemented (20–22).

The GRASP requires participants to engage in 60 min of daily exercise, monitored weekly by therapists. The program comprises three exercise levels (Levels 1, 2, and 3), correlating to severe, moderate, and mild impairment levels, respectively, according to the Fugl–Meyer injury scale.

To develop a rehabilitation exercise regimen, we combined exercises from Levels 2 and 3 of the GRASP, integrating elements from the rehabilitation exercise program of the local hospital. These exercises encompass movements and coordination exercises for various UL joints, along with activities targeting hand function and daily life tasks. Through these exercises, we aim to promote UL functional recovery, enhance the self-care abilities of patients, and improve their quality of life.

The specific rehabilitation training content was as follows:

(a) Shoulder training: The patient sat, and the training was divided into six sub-movements: (1) shoulder abduction, (2) shoulder adduction, (3) horizontal shoulder abduction, (4) shoulder horizontal adduction, (5) external shoulder, and (6) internal shoulder rotation. Each sub-action lasted for 5 s, followed by 15 s of rest after the action was restored, and the training cycle set was performed 20 times.
(b) Elbow training: The patient sat, and the training was divided into four sub-movements: (1) arm bending, (2) arm straightening, (3) arm valgus, and (4) arm varus. Rest was allowed for 15 s after the action was restored, and the action set was repeated 20 times.
(c) Wrist training: The patient adopted a sitting position, and the training was divided into four sub-actions: (1) wrist joint bending, (2) wrist joint extension, (3) radial deviation, and (4) ulnar deviation. Each sub-action lasted for 5 s, with an interval of 15 s. This set of movements was repeated five times.

### 2.2. Participants

From October 2022 to April 2023, a total of 12 community patients with stroke (five females and seven males; mean age 56.25 ± 7.25) were recruited to participate in rehabilitation training. Since the rehabilitation training content is derived from GRASP Levels 2 and 3, it corresponds to mild impairment according to the Fugl–Meyer injury scale. Therefore, the inclusion criteria for stroke participants in our study are as follows: (1) first unilateral stroke ≥2 weeks after stroke confirmed by computed tomography or magnetic resonance imaging; (2) Fugl–Meyer UL assessment (FMA-UE) >36 points and hand movement score >7; (3) basic mental state examination score ≥25; and (4) able to sit independently on a chair for at least 1 h. The exclusion criteria were as follows: (1) unilateral neglect or vision problems, (2) hypersensitivity to the electrode gel, and (3) receiving noninvasive brain stimulation during the study. All participants completed a cardiopulmonary exercise test (CPET) and received physician clearance before starting the intervention.

The 12 participants involved in the study exhibit mild impairment in their ULs, with Brunnstrom scores ranging between Level 4 and Level 6. They do not present any additional disabilities, possess basic activities of daily living, and do not show signs of post-stroke depression. These stroke survivors are currently primarily focused on rehabilitating fine motor skills, enhancing coordination, and improving movement speed and accuracy.

This study was approved by the Ethics Committee of First Hospital of Jilin University (2022-514) and conducted in accordance with the Declaration of Helsinki. An experienced therapist who was blinded to the study performed all clinical measurements. The FMA-UE is a classic motor measurement scale designed for UL hemiplegia (23) that reflects UL motor impairment severity (Table 1).

**Table 1:** Basic information and clinical characteristics of participants.

Participants information										
Subject	Gender	Age	Weeks since stroke	Affected arm	Dominant hand	FMA-UE				
						Arm (Max = 36)	Wrist (Max = 10)	Hand (Max = 14)	Coordination (Max = 6)	Total (Max = 66)
01	Male	49	24	Right	Right	33	6	12	4	55
02	Male	53	23	Left	Right	31	5	11	5	52
03	Male	55	13	Right	Right	31	6	11	2	50
04	Male	55	20	Right	Right	30	7	9	3	49
05	Male	57	12	Left	Right	22	7	9	4	42
06	Male	65	19	Right	Right	29	5	12	4	50
07	Male	66	19	Right	Right	17	6	13	4	40
08	Female	51	17	Right	Right	29	7	11	3	50
09	Female	52	12	Left	Right	21	5	8	4	38
10	Female	54	14	Left	Right	29	6	10	5	50
11	Female	56	12	Left	Right	34	6	14	5	59
12	Female	62	12	Right	Right	26	7	9	2	44
Mean		56.25	16.42			27.67	6.08	10.75	3.75	48.25

### 2.3. Experimental setup

#### 2.3.1. EEG and EMG acquisition

**Figure 1:**
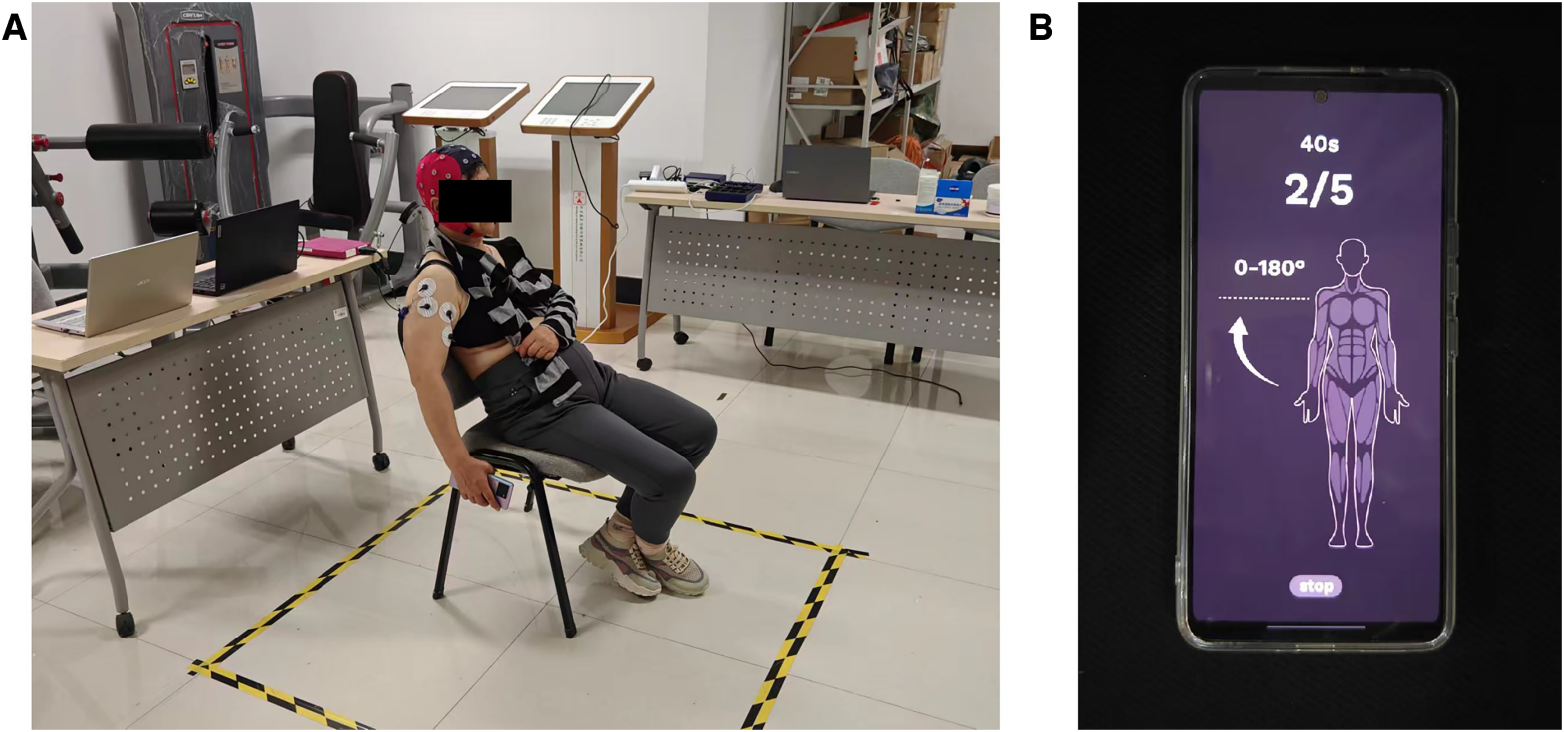
(**A**) Experiment environment. (**B**) Experiment material.

Figure 1 shows the experimental setup and environment used in this study. The experiment was carried out in a quiet environment, with each subject sitting comfortably on an armless chair, and the experimenters put EEG caps and wireless surface electromyography onto the participants.

The 32-channel ANT neuro eego™ mylab product was used to collect the EEG signals (C3, C4, CP1, CP2) of each channel, of which C3 and C4 corresponded to the body motor center in the precentral gyrus of the frontal lobe and CP1 and CP2 corresponded to the body motor center in the postcentral gyrus of the frontal lobe, with a sampling rate of 1,000 Hz. The eight-channel MYON Wireless EMG was used to record the corresponding EMG signals, with a sampling rate of 1,000 Hz.

The locations of EMG signal acquisition in the different sub-experiments differed. Shoulder rehabilitation training mainly collected EMG signals of the deltoid (DEL), supraspinatus (SSP), biceps brachii (BB), and triceps brachii (TB); elbow rehabilitation training mainly collected signals from the brachioradialis (BRD), brachialis (BR), BB, and TB; wrist rehabilitation mainly included the flexor carpi ulnaris (FCU), extensor carpi ulnaris (ECU), flexor carpi radialis (FCR), and extensor carpi radialis (ECR).

In this experiment, four channels (emg0, emg1, emg2, emg3) were turned on, and four pairs of electrodes were attached to the skin surface of the abdomen of the relevant muscles, with the centers separated by 2 cm. The impedance of all EEG and EMG electrodes was kept below 10 kΩ. Synchronized with a sampling frequency of 1,000 Hz, the event time was marked using E-prime 3.0.

#### 2.3.2. Experimental scheme

Due to the prolonged duration of the rehabilitation exercises, conducting consecutive sessions may potentially influence the experimental outcomes. Therefore, to prevent any sequence effects, the study is designed in a manner where each participant is randomly assigned to either the game-assisted rehabilitation group (G) or the conventional rehabilitation group (R) in the first session. Following a random number of days *N* (where 7 ≤ *N* ≤ 14) after the initial session, the second session takes place, and the experimental groups are interchanged (G→R or R→G). This approach ensures that each participant undergoes both forms of rehabilitation exercises, allowing for a more comprehensive evaluation of the efficacy and impact of the two rehabilitation methods. The two groups had the same motor tasks, and the patients in the game-assisted rehabilitation group underwent rehabilitation training under the instructions of the game and somatosensory feedback while holding the mobile phone. The patients in the conventional rehabilitation group followed the rehabilitation training instructions given by the staff.

**Figure 2:**
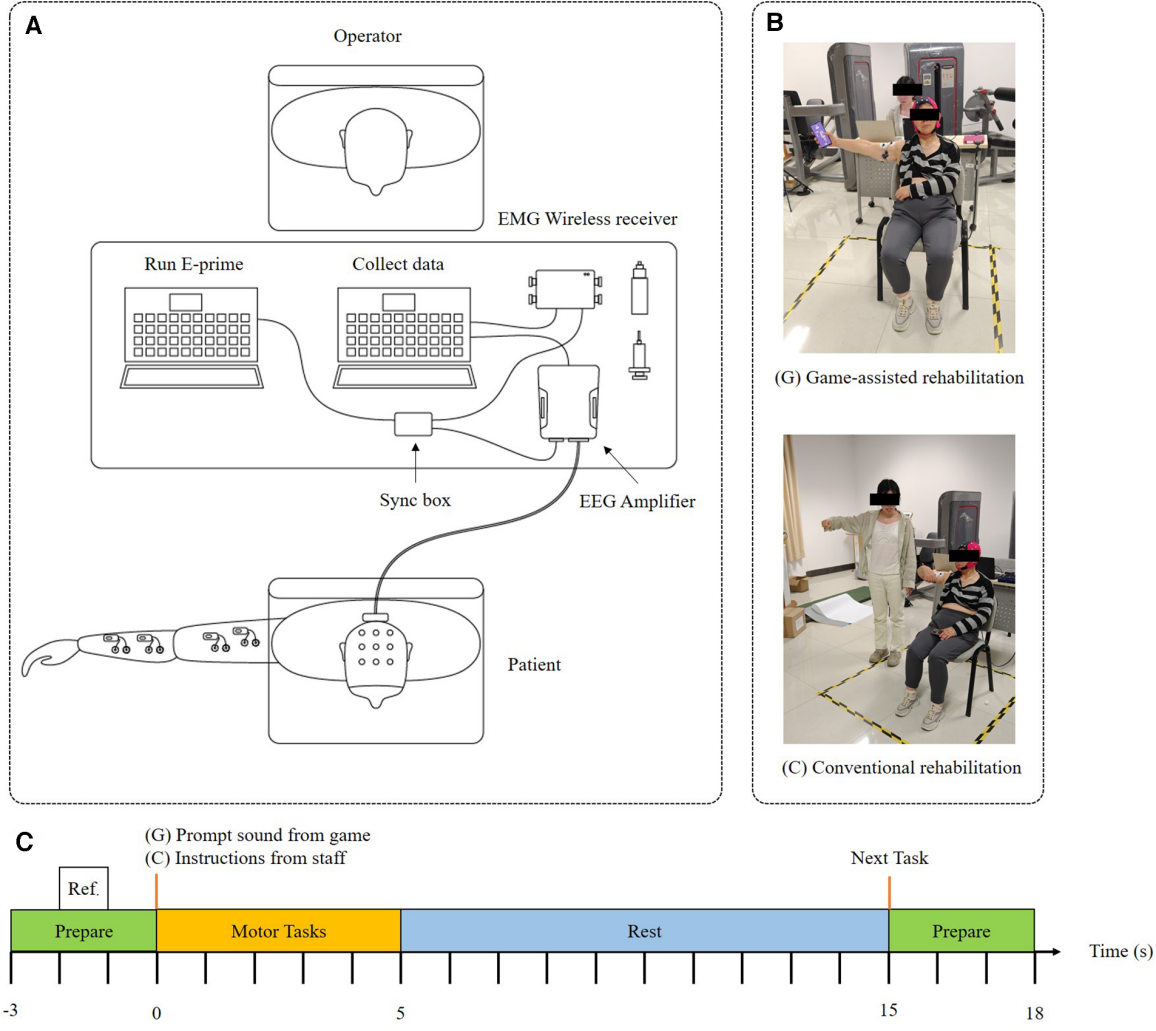
(**A**) System setup. (**B**) Experimental group. (**C**) Trial description.

All motor tasks were divided into a preparation phase (S1), movement phase (S2), and rest phase (S3) to reduce the mutual influence before and after the task. Taking the instructions of the game/staff as the 0 s of the time scale, the arrangement of each motor task was as follows: −3 to 0 s was the preparation stage (S1), which provided the reference measurement in the idle state before the motor task; 0–5 s was the exercise phase (S2), with each sub-action MA classified as a single trial and repeated 20 times to reduce errors during the test; 5–15 s was the rest phase (S3), which calculated the influence of the current MA; and 15–18 s was the preparation stage (S1) for the next motor task. The baseline was set from −2 s to −1 s to prevent the signal from being polluted by the response to the previous stimulus, as shown in Figure 2.

The motor task was specifically designed such that the participant performed 14 sub-actions (a1, a2, a3, a4, a5, a6, b1, b2, b3, b4, c1, c2, c3, c4) in shoulder, elbow, and wrist training. The order of the sub-actions that each patient needed to perform was randomly disrupted, and there was a 10-min rest period after each sub-action was completed. A total of 12 total patients with stroke participated in the two experimental groups. Before the experiment officially started, each subject stood still for 5 min, and the subjects started to attempt the motor tasks after receiving instructions.

### 2.4. Signal processing and data analysis

#### 2.4.1. EEG processing and analysis

The EEG signals recorded in the channel were preprocessed in a series of steps. The data analysis was performed offline in MATLAB (R2019a) using the EEGLAB toolbox. The EEG recordings were re-referenced to the average value of channels TP9 and TP10, and basic finite impulse response filtering was carried out with a 30 Hz low-pass and 0.1 Hz high-pass filter, respectively. Segments with low signal-to-noise ratio were rejected, and independent component analysis was implemented using the “pop_runica” function from EEGLAB (24). Artifacts such as eye movements, electromyography, and sweat were corrected using ADJUST 1.1.1. The EEG data were segmented from 300 ms before stimulus S2 to 1,500 ms after the stimulus onset, with the period from 200 to 100 ms before as the “pre-event” reference baseline. The segments were classified based on conditions. Trials with no behavioral responses, amplifier saturation, muscle burst activity, or peak-to-peak deviation exceeding ±80 µV were excluded from the experiment (25).

The steps included (1) import data; (2) electrode positioning; (3) remove unnecessary electrodes; (4) re-reference; (5) 0.01–30 Hz band-pass filtering; (6) segmentation and baseline correction; (7) run ICA; (8) remove electrooculopathy and artifacts; and (9) resample to 500 Hz.

Only the higher alpha (8–13 Hz) and beta (14–30 Hz) frequency ranges were analyzed because motor preparation and execution generate ERD in the sensorimotor regions at 10 and 20 Hz. The time-frequency distribution of the EEG was estimated using a windowed Fourier transform (WFT) (26) with a fixed 200 ms Hanning window. For each trial, the WFT produced a complex time-frequency estimate F(t, F) at each time-frequency point (*t*, *F*) from −3,000 to 15,000 ms (in steps of 5 ms) in the time domain, 1–30 Hz (in 1 Hz steps) in the frequency domain, and a power spectrum *P*, *P*(*t*, *f*) = | *f* (*t*, *f*)|. We calculated the power spectrum of all channels at alpha (8–13 Hz) and beta (14–30 Hz) to determine the ERD and ERS of the motor task by the affected UL. The ERD/ERS transition was defined as the percentage decrease/increase in instantaneous power density at the “event” compared to the “pre-event” baseline value (27).

$$\;\mathrm{ERD}/\;\mathrm{ERS}=\frac{\;A-\;R}{\;R}\times 100\text{\%}$$

#### 2.4.2. EMG processing and analysis

The sEMG signal is a bioelectrical current generated by the contraction of surface muscles. The nervous system controls muscle activity—contraction or relaxation—resulting in diverse signals from different muscle fiber motor units in the skin at any given time. sEMG is a non-stationary microelectrical signal with amplitudes ranging from 0 to 1.5 mV, containing pertinent information between 20 and 200 Hz (28). During signal acquisition, interference occurs from the 50 Hz power frequency signal and low-frequency signals below 20 Hz, hence the selection of Butterworth high-pass and band-pass filters for sEMG to filter and remove noise (29). In this experiment, the sEMG was segmented using a sliding window method to acquire several sEMG sub-data segments for subsequent feature computation and analysis. This involved extracting signal characteristics with a sliding window of a sample to ensure signal continuity (30).

Maximum voluntary contraction percentage (MVC%) is a commonly used index for evaluating EMG signals because individual differences have little influence on EMG signals. However, obtaining MVC% is cumbersome and requires participants to repeat maximal isometric contractions, which is not suitable for special populations, such as patients with hemiplegia. The root mean square (RMS) is a time domain index of EMG signals that reflects the muscle load level in real time and measures the load on the participant during the measurement period. It has the advantages of easy operation and real-time response (31); therefore, the RMS value was chosen as the measurement index. The collected raw EMG data were band-pass filtered in the 10–200 Hz band to remove 50 Hz industrial frequency interference and then corrected.

The total number of myoelectric data sample points filtered by each motor task is *N*, the myoelectric signal data after filtering and segmentation of each sample channel is Data, and the RMS is calculated as

$$\;\mathrm{RMS}=\sqrt{\frac{\overset{\;N}{\underset{\;i=0}{\sum }}\;\mathrm{Data}{\left[\;i\right]}^{2}}{\;N}}$$

#### 2.4.3. Statistical analysis

Statistical analysis was performed using SPSS (IBM). Due to the small sample size, the data were confirmed to have a normal distribution according to the Shapiro–Wilk normality test. Because each subject participated in the game-assisted rehabilitation experiment and the conventional rehabilitation experiment (C), there was no individual difference between the two experiments for the same subject. Therefore, the Wilcoxon signed-rank test was used for the between-group analysis to determine whether the *p*-value of paired samples in each group was significant (*p* < 0.05).

## 3. Results

### 3.1. Results of shoulder rehabilitation training

#### 3.1.1. ERD comparison of shoulder rehabilitation training

**Figure 3:**
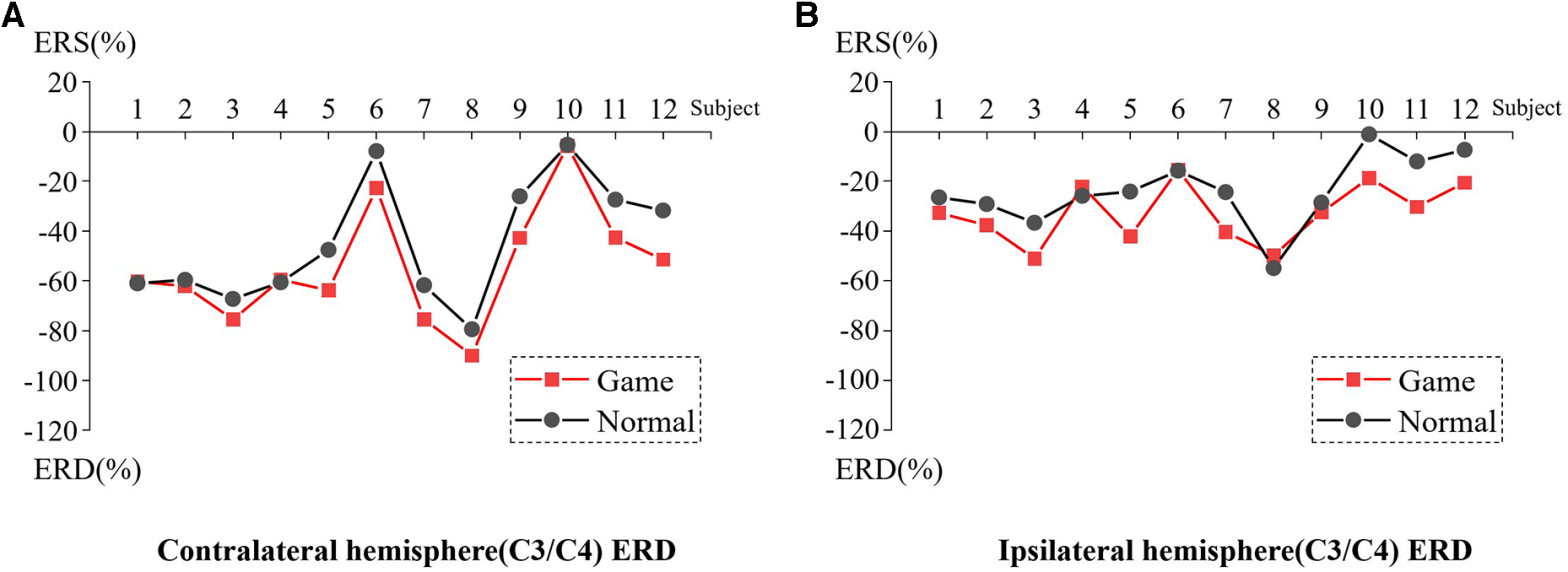
(**A**) ERD of Contralateral hemisphere(C3/C4) during shoulder rehabilitation training. (**B**) ERD of Ipsilateral hemisphere(C3/C4) during shoulder rehabilitation training.

Figure 3 presents the ERD values of the contralateral and ipsilateral hemisphere motor perception areas in the game-assisted and conventional rehabilitation.

The results showed that during the execution of the motor task, the ERD value of the contralateral hemisphere motion perception area in the game-assisted rehabilitation group (*M* = −54.300, SD = 23.371) was higher than that in the conventional rehabilitation group (*M* = −44.616, SD = 24.266) and had a larger magnitude (*M* = −9.685, SD = 7.553). The paired-sample Wilcoxon signed-rank test was performed on the two groups of samples. Based on the variable game-assisted rehabilitation ERD paired with conventional rehabilitation ERD, Cohen's *d* value of the difference was 0.407 (small). The *p*-value was 0.008***, which was significant at the level and rejected the null hypothesis. Therefore, there was a significant difference in the ERD of the contralateral hemisphere between the two groups in the (a) shoulder training experiment.

During the execution of the motor task, the ERD value of the motion perception area of the ipsilateral hemisphere (*M* = −32.719, SD = 11.934) under game-assisted rehabilitation (G) was compared with the ERD value (*M* = −23.830, SD = 14.086) of a larger magnitude (*M* = −8.889, SD = 8.620). The paired-sample Wilcoxon signed-rank test was performed on the two groups of samples. Based on the variable game-assisted rehabilitation ERD paired with conventional rehabilitation ERD, Cohen's *d* value of the difference was 0.681 (medium). The *p*-value was 0.012**, which was significant at the level and rejected the null hypothesis. Therefore, there was a significant difference in the ERD of the ipsilateral hemisphere between the two groups in the shoulder training experiment.

#### 3.1.2. RMS comparison of shoulder rehabilitation training

**Figure 4:**
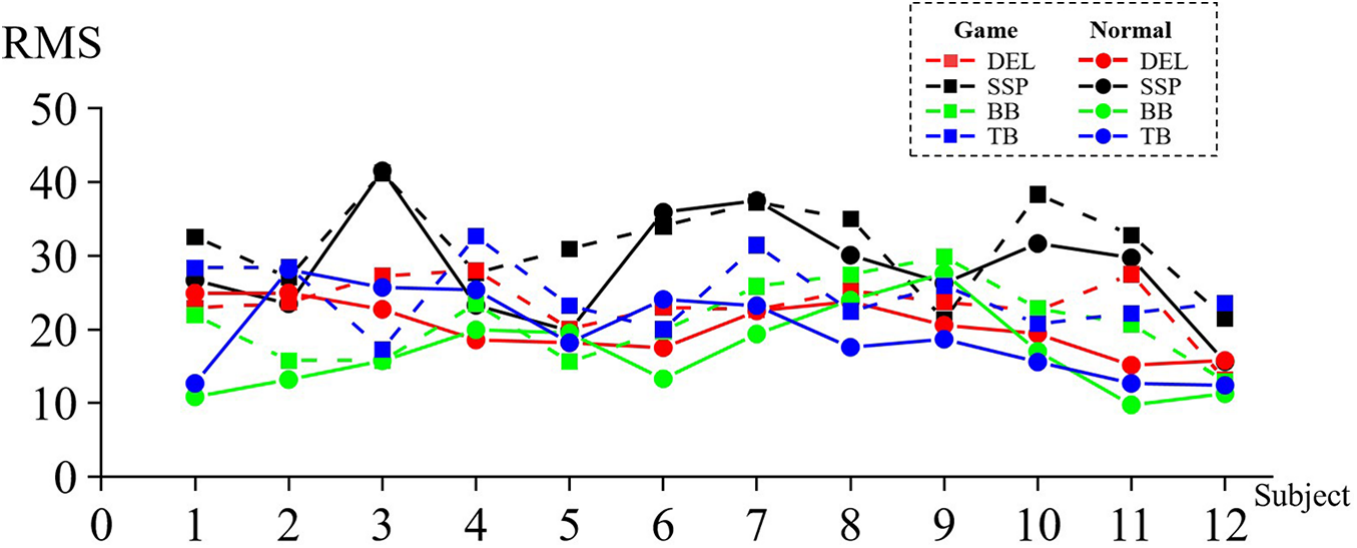
RMS value of the corresponding muscle during shoulder rehabilitation training.

Figure 4 shows the RMS value of the affected side in game rehabilitation assistance when 12 patients underwent motor task [DEL (*M* = 31.661, SD = 6.284), SSP (*M* = 23.317, SD = 3.971), BB (*M* = 21.006, SD = 5.252), and TB (*M* = 24.708, SD = 4.718)] and conventional rehabilitation (C) [DEL (*M* = 28.461, SD = 7.529), SSP (*M* = 20.336, SD = 3.422), BB (*M* = 16.792, SD = 5.515), and TB (*M* = 19.514, SD = 5.620)]. The four pairs of muscles had larger RMS values under the game-assisted rehabilitation condition than under the conventional rehabilitation condition. The differences were DEL (*M* = 3.200, SD = 4.381), SSP (*M* = 2.981, SD = 4.511), BB (*M* = 4.214, SD = 4.292), and TB (*M* = 5.195, SD = 6.592).

Two groups of four pairs of muscle RMS samples were subjected to a paired-sample Wilcoxon signed-rank test based on the variable RMS of game-assisted rehabilitation muscles paired with the RMS of conventional rehabilitation muscles. Cohen's *d* values of DEL, SSP, BB, and TB were 0.462 (small), 0.804 (medium), 0.782 (medium), and 1.001 (very large), and their *p*-values were 0.041** (significant), 0.041** (significant), 0.012** (significant), and 0.028** (significant), respectively. Therefore, there was a significant difference in the RMS between the two groups of muscles in the shoulder training experiment.

### 3.2. Results of elbow rehabilitation training

#### 3.2.1. ERD comparison of elbow rehabilitation training

**Figure 5:**
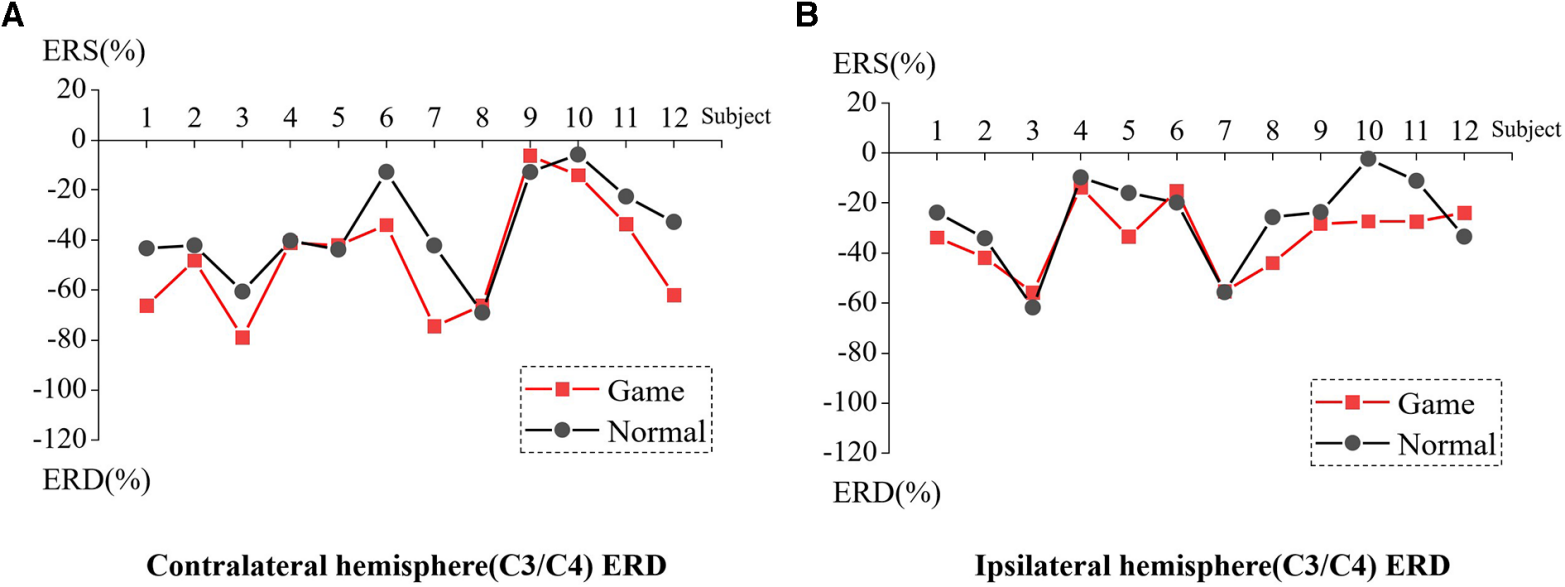
(**A**) ERD of Contralateral hemisphere(C3/C4) during elbow rehabilitation training. (**B**) ERD of Ipsilateral hemisphere(C3/C4) during elbow rehabilitation training.

Figure 5 presents the ERD values of the contralateral and ipsilateral hemisphere motor perception areas in the game-assisted and conventional rehabilitation.

The results showed that during the execution of the motor task, the ERD value of the contralateral hemisphere motion perception area in the game-assisted rehabilitation group (*M* = −47.223, SD = 23.122) was higher than that in the conventional rehabilitation group (*M* = −35.632, SD = 19.271) and had a larger magnitude (*M* = −11.591, SD = 13.046). The paired-sample Wilcoxon signed-rank test was performed on the two groups of samples. Based on the variable game-assisted rehabilitation ERD paired with conventional rehabilitation ERD, Cohen's *d* value of the difference was 0.545 (medium). The *p*-value was 0.023**, which was significant and rejected the null hypothesis. Therefore, there was a significant difference in the ERD of the contralateral hemisphere between the two groups in (b) elbow training experiment.

During the execution of the motor task, the ERD value of the motion perception area of the ipsilateral hemisphere (*M* = −33.318, SD = 13.735) under the condition of game-assisted rehabilitation (G) was compared with the ERD value (*M* = −26.372, SD = 17.766) of a larger magnitude (*M* = −6.947, SD = 10.906). The paired-sample Wilcoxon signed-rank test was performed on the two groups of samples. Based on the variable game-assisted rehabilitation ERD paired with conventional rehabilitation ERD, Cohen's *d* value of the difference was 0.437 (small). The *p*-value was 0.071*, which reflected no statistical significance, and the null hypothesis was not rejected. Therefore, there was no significant difference in the ERD of the ipsilateral hemisphere between the two groups in the elbow training experiment.

#### 3.2.2. RMS comparison of elbow rehabilitation training

**Figure 6:**
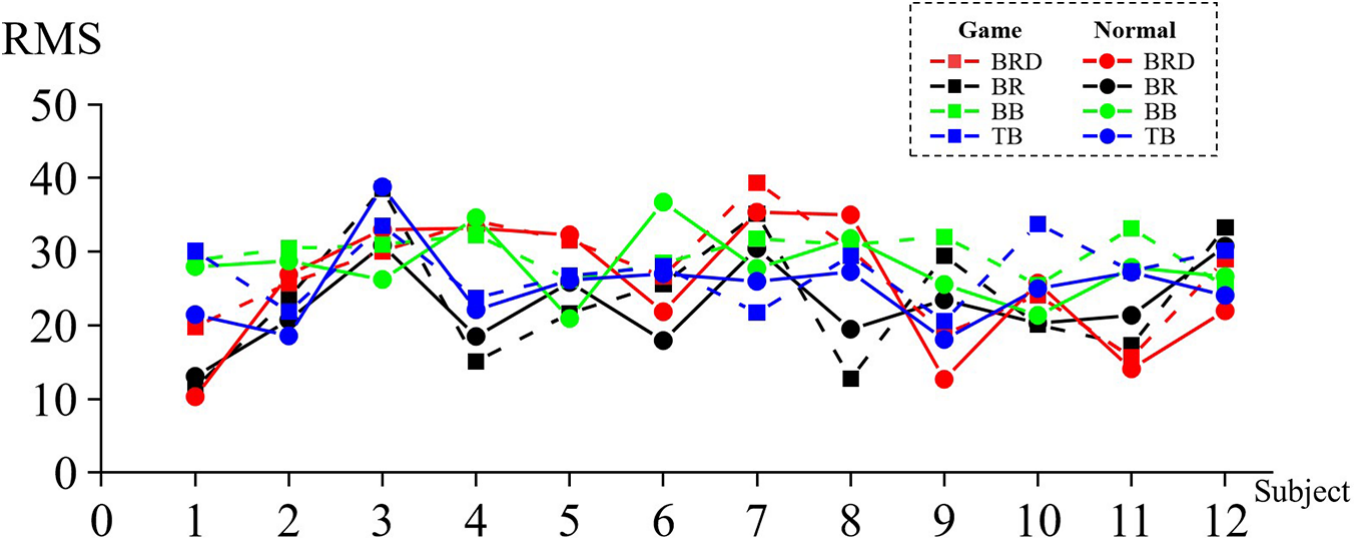
RMS value of the corresponding muscle during elbow rehabilitation training.

Figure 6 shows the RMS values of the affected side of game rehabilitation assistance (G) for 12 patients who underwent motor task [BRD (*M* = 23.678, SD = 8.960), BR (*M* = 27.103, SD = 6.800), BB (*M* = 27.247, SD = 4.485), and TB (*M* = 27.247, SD = 4.485)] and conventional rehabilitation (C) [BRD (*M* = 22.717, SD = 5.698), BR (*M* = 25.191 SD = 9.058), BB (*M* = 28.008, SD = 4.676), and TB (*M* = 25.135, SD = 5.381)].

The four pairs of muscles had larger RMS values under the game-assisted rehabilitation condition than under the conventional rehabilitation condition. The differences were BRD (*M* = 0.961, SD = 5.008), BR (*M* = 1.912, SD = 4.380), BB (*M* = 1.653, SD = 4.276), and TB (*M* = 2.112, SD = 4.346).

Two groups of four pairs of muscle RMS samples were subjected to a paired-sample Wilcoxon signed-rank test based on the variable RMS of game-assisted rehabilitation muscles, paired with the RMS of conventional rehabilitation muscles. Cohen's *d* values of BRD, BR, BB, and TB were 0.128 (very small), 0.239 (quite small), 0.430 (quite small), and 0.426 (quite small), respectively, and their *p*-values were 0.53, 0.209, 0.158, and 0.084*, respectively. Thus, there were no statistically significant differences at any level, which reflects a lack of statistically significant differences in RMS between the two groups of muscles in the elbow training experiment.

### 3.3. Results of wrist rehabilitation training

#### 3.3.1. ERD comparison of wrist rehabilitation training

**Figure 7:**
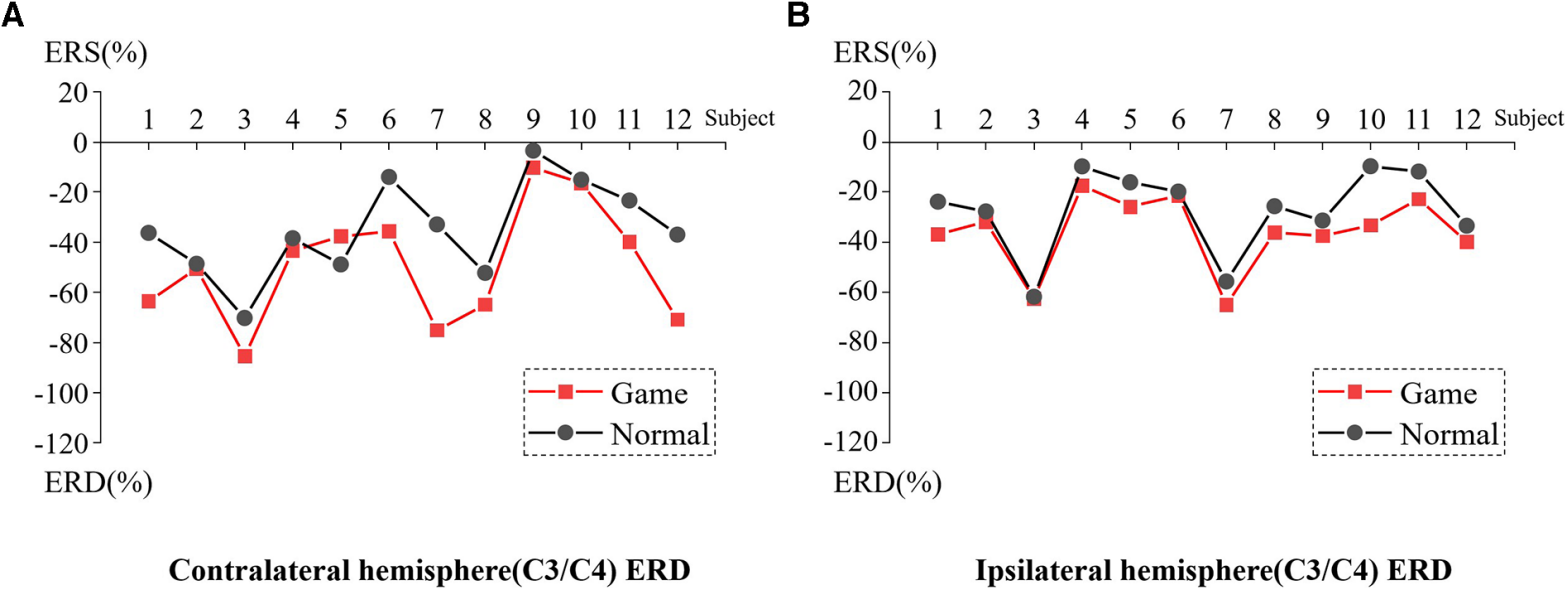
(**A**) ERD of Contralateral hemisphere(C3/C4) during wrist rehabilitation training. (**B**) ERD of Ipsilateral hemisphere(C3/C4) during wrist rehabilitation training.

Figure 7 presents the ERD values of the contralateral and ipsilateral hemisphere motor perception areas in the game-assisted and conventional rehabilitation.

The results showed that during the execution of the motor task, the ERD value of the contralateral hemisphere motion perception area in the game-assisted rehabilitation group (*M* = −49.428, SD = 23.235) was higher than that in the conventional rehabilitation group (*M* = −35.003, SD = 18.839) and had a larger magnitude (*M* = −14.425, SD = 15.069). The paired-sample Wilcoxon signed-rank test was performed on the two groups of samples. Based on the variable game-assisted rehabilitation ERD paired with conventional rehabilitation ERD, Cohen's *d* value of the difference was 0.682 (medium). The *p*-value was 0.008***, which was significant and rejected the null hypothesis. Therefore, there was a significant difference in the ERD of the contralateral hemisphere between the two groups in the wrist training experiment.

During the execution of the motor task, the ERD value of the motion perception area of the ipsilateral hemisphere (*M* = −35.834, SD = 14.863) under the condition of game-assisted rehabilitation was compared with the ERD value (*M* = −27.180, SD = 16.783) with a larger magnitude (*M* = −8.655, SD = 5.999). The paired-sample Wilcoxon signed-rank test was performed on the two groups of samples. Based on the variable game-assisted rehabilitation ERD paired with conventional rehabilitation ERD, Cohen's *d* value of the difference was 0.546 (medium). The *p*-value was 0.002***, which was significant and rejected the null hypothesis. Therefore, there was a significant difference in the ERD of the ipsilateral hemisphere between the two groups in the wrist training experiment.

#### 3.3.2. RMS comparison of wrist rehabilitation training

**Figure 8:**
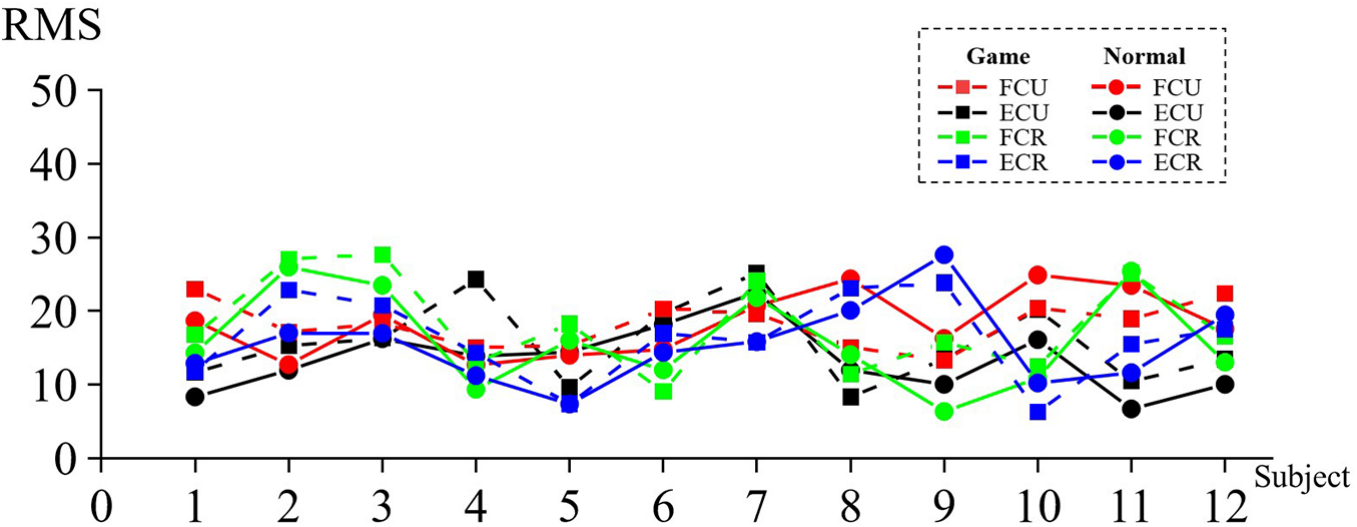
RMS value of the corresponding muscle during wrist training.

Figure 8 shows the RMS values of the affected side of game rehabilitation assistance (G) for 12 patients who attempted motor task [FCU (*M* = 16.699, SD = 6.302), ECU (*M* = 18.218, SD = 3.102), FCR (*M* = 18.097, SD = 6.405), and ECR (*M* = 16.333, SD = 5.832)] and conventional rehabilitation (C) [FCU (*M* = 14.439, SD = 5.140), BR (*M* = 18.286, SD = 4.396), FCR (*M* = 16.041, SD = 6.564), and ECR (*M* = 15.377, SD = 5.433)].

The four pairs of muscles had larger RMS values in the game-assisted rehabilitation condition than in the conventional rehabilitation condition, and the differences were FCU (*M* = 2.260, SD = 3.893), ECU (*M* = −0.068, SD = 4.640), FCR (*M* = 2.056, SD = 3.266), and ECR (*M* = 0.956, SD = 3.207).

Two groups of four pairs of muscle RMS samples were subjected to a paired-sample Wilcoxon signed-rank test based on the variable RMS of game-assisted rehabilitation muscles paired with the RMS of conventional rehabilitation muscles. Cohen's *d* values of FCU, ECU, FCR, and ECR were 0.362 (quite small), 0.018 (very small), 0.317 (quite small), and 0.170 (very small), respectively, and their *p*-values were 0.117, 0.875, 0.071*, and 0.388, respectively. There were no statistically significant differences at any levels; therefore, there was no significant difference in RMS between the two groups of muscles in the (c) wrist training experiment.

## 4. Discussion

Using technology and design to assist in the rehabilitation of hemiplegia after stroke is an important issue in the health field. This study used EEG and EMG to explore the possibility of serious games assisting the rehabilitation of UL hemiplegia after stroke. In the experiment, 12 patients with UL hemiplegia attempted motor tasks under conditions of serious game-assisted rehabilitation and conventional rehabilitation. We collected and compared the EEG and EMG data.

**Figure 9:**
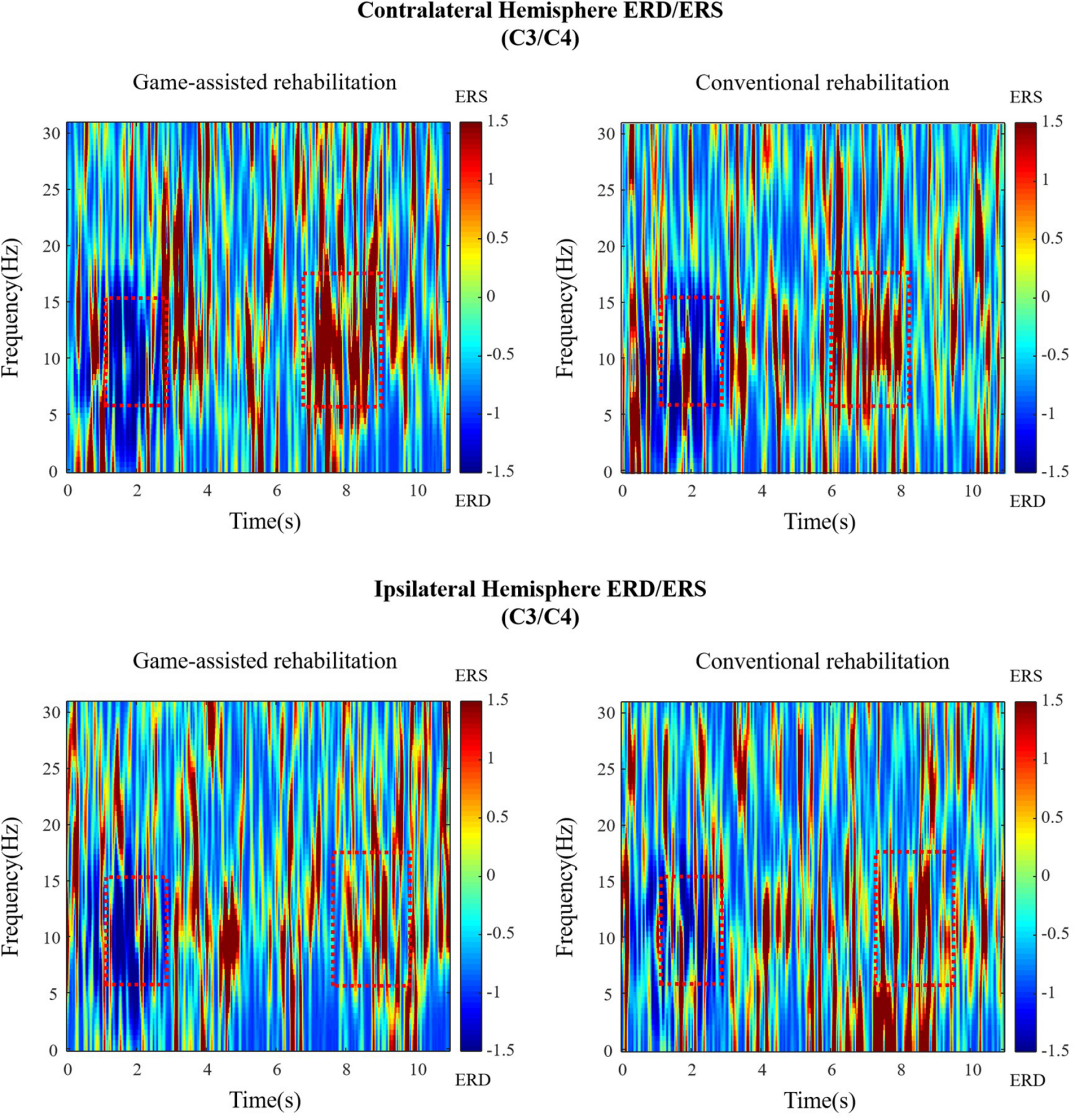
The time-frequency diagram of Subject 1 during shoulder rehabilitation training. Blue represents ERD, red represents ERS, and the red rectangle represents the approximate time and frequency.

The research primarily compares the differences between the two experiments. Thus, the EEG section selected the motor perception areas (C3, C4, CP1, CP2) for primary analysis, while the EMG section chose the main muscles for each training group. As the data reflect only local features and are influenced by complex factors, comparisons between the data of different subjects do not lead to valid conclusions. However, for individual subjects, the variables from the two experiments have been controlled, enabling a comparison of EEG and EMG changes after serious game-based rehabilitation interventions (as shown in Figure 9).

### 4.1. ERD differences in serious game-assisted rehabilitation

From the experimental results, in the MA tasks of the three rehabilitation training, the motion perception area of the contralateral hemisphere of patients had a stronger ERD in game-assisted rehabilitation than in conventional rehabilitation, and the results were statistically significant. This finding suggests a better activation of the sensorimotor areas of the brain of the patient during the use of serious gaming applications (32). Tactile stimulation results in greater motor-related cortical activation in the alpha–beta band (33); therefore, we speculate that the vibration feedback function in the serious game app has played a role in motor cortex activation. Simultaneously, the serious game app provides vibration signals of different strengths and weaknesses according to the rhythm of the action, providing supplements to the limb perception of the patient.

Sensorimotor areas of the ipsilateral hemisphere also had stronger ERD in game-assisted rehabilitation compared to that in conventional rehabilitation, with significant differences in shoulder training and wrist training but not in elbow training. This result was expected because ERD is generally more severe in the contralateral hemisphere than in the ipsilateral hemisphere during MA tasks (34).

Three horizontal comparisons of shoulder, elbow, and wrist training showed that the ERD intensity difference between shoulder training in game-assisted rehabilitation and conventional rehabilitation was the strongest of the three exercises; that is, the serious game app played the largest role in the activation of the motor cortex in shoulder rehabilitation.

During the experiment, we found that the patients with hemiplegia observed their rehabilitation movements very carefully during elbow and wrist training. The two training movements had a small range of motion and were observed within range of sight. In mirror visual feedback, vision is often used as an information supplement for limb perception impairments (35). Therefore, we assumed that in elbow training and wrist training, the observation of the actions of the patient also played a role in the activation of the motor cortex, and the supplementation of visual signals diluted the gain effect brought about by the vibration feedback of the serious game application.

In addition, the voice broadcast function provides auditory stimulation, and the rhythm and instructions of the movement provide patients with supervision and guidance, which encourages them to have a stronger willingness to perform the actions.

### 4.2. RMS differences in serious game-assisted rehabilitation

From the experimental results, among the three MA rehabilitation training tasks, the RMS of patients with hemiplegia during game-assisted rehabilitation was greater than that during conventional rehabilitation, except for one MA task. However, after the paired-sample Wilcoxon signed-rank test, the RMS significantly differed only for shoulder training. This finding suggests that game-assisted rehabilitation is more intense than conventional rehabilitation in terms of the actual muscle motor performance, although the difference was not statistically significant. Simultaneously, in elbow and wrist training, the patient observed his or her own movements, and the visual feedback encouraged the patient to complete the MA task more properly; therefore, the difference in RMS between the two rehabilitation trainings was small.

### 4.3. ERD and RMS in rehabilitation training

RMS amplitude represents the degree of muscle activity. The difference between the experimental conditions of the G and C groups was more obvious in the motor cortex activity but less in terms of the muscle activity change. The ERD in the bilateral hemispheres, ipsilateral hemispheres, contralateral hemispheres, and C3/C4 channels in patients with hemiplegia did not significantly differ between the MA and MI tasks (36). In active movements, MA-ERD has been greatly affected by the subjective exercise willingness of a patient (37). Therefore, the vibration feedback and voice broadcast functions of the serious game rehabilitation application can enhance the willingness of a patient to exercise. According to the results, the serious game rehabilitation app can play a certain role in activating the motor cortex of the brain of the patient but does not directly improve muscle activity.

### 4.4. Feasibility of serious game rehabilitation

This experiment did not observe the rehabilitation changes of the two groups from the complete rehabilitation cycle but rather conducted a short-term comparison of the activation degrees of the cerebral motor cortex and of the exercise muscles during the rehabilitation process, to predict the feasibility of the serious game rehabilitation app. Under the condition of game-assisted rehabilitation, the stronger cerebral motor cortex signal of patients confirmed the feasibility of the serious game app in rehabilitation assistance, to a certain extent. Because the activity intensity of the muscles depends more on objective physical fitness and the actual degree of rehabilitation, it is difficult to make a huge change in a short period. Therefore, it is foreseeable that serious game rehabilitation apps cannot significantly improve muscle activity.

### 4.5. Factors that may influence experimental results

Considering that each participant underwent the first and second experiments on different days, numerous factors might have influenced the measurements of brain activity. These factors include environmental elements such as variations in temperature and humidity within the 2 days, potentially affecting the brain activity of the participants during the experiments. Moreover, the emotional state of the participants could also be impacted, such as the influence of short-term events, leading to different emotional states during the two experiments, thereby affecting the measurement of brain activity. In addition, the medication and sleep status of the participants on the day of the experiment could also influence the measurement of brain activity.

Factors influencing EMG signals are similarly complex. Environmental elements, such as variations in temperature and humidity within the 2 days, might impact the muscle activity and endurance of participants during the experiments. Variances in physiological states, emotional conditions, and pain levels may also affect EMG data. Furthermore, the initial state of the muscles and subtle shifts in electrode placement between the two experiments could impact data accuracy.

## 5. Limitations

In our study, we only conducted comparative trials of the two rehabilitation methods on the same participants without long-term clinical observation and recording. In addition, the limited number of participants and experimental sessions restricted our ability to comprehensively assess the effectiveness of game-assisted rehabilitation. To gain a better understanding of the potential of game-assisted rehabilitation, we recommend increasing the number of participants and experimental sessions in future research. Long-term clinical observation and recording should be incorporated. Moreover, further improvements in experimental design and methodology are essential to enhance the reliability and accuracy of the experiments.

## Data Availability

The original contributions presented in the study are included in the article/Supplementary Material, further inquiries can be directed to the corresponding author.
